# Alternation of the gut microbiota in metabolically healthy obesity: An integrated multiomics analysis

**DOI:** 10.3389/fcimb.2022.1012028

**Published:** 2022-11-01

**Authors:** Han Chen, Nana Tang, Qiang Ye, Xin Yu, Ruoyun Yang, Hong Cheng, Guoxin Zhang, Xiaoying Zhou

**Affiliations:** ^1^ Department of Gastroenterology, The First Affiliated Hospital of Nanjing Medical University, Nanjing, China; ^2^ The First Clinical Medical College, Nanjing Medical University, Nanjing, China; ^3^ Department of Neurology, The First Affiliated Hospital of Nanjing Medical University, Nanjing, China

**Keywords:** metabolically healthy obesity, gut microbiota, GMrepo database, metagenomic analysis, metabonomic analysis, diet-induced obesity

## Abstract

**Background:**

Although the gut microbiota may be involved in obesity onset and progression, the exact association of the gut microbiota in metabolically healthy obesity (MHO) remains largely unknown.

**Methods:**

An integrated paired-sample metagenomic analysis was conducted to investigate the gut microbial network and biomarkers of microbial species from the MHO and healthy non-obese subjects in the GMrepo database. Further explorations were performed in the MHO mice model using a multiomics analysis to detect changes in the composition and function of the intestinal microbiome and associated metabolites.

**Results:**

In the human study, 314 matched metagenomic data were qualified for the final analysis. We identified seven significantly changed species possibly involved in MHO pathogenesis (MHO-enriched: *Bacteroides vulgatus*, *Megamonas sp*; MHO-depleted: *Butyrivibrio crossotus*, *Faecalibacterium prausnitzii*, *Bacteroides cellulosilyticus*; *Eubacterium siraeum*; *Bacteroides massiliensis*). In the murine study, we found 79 significantly-changed species which may have possible associations with the MHO phenotype. The depletion of *Bacteroides cellulosilyticus* was commonly recognized in the human and murine MHO phenotype. Consistent with the metagenomic data, liquid chromatography-mass spectrometry (LC/MS) revealed significantly changed gut metabolites, which may promote MHO pathogenesis by altering the amino acids and lipid metabolic pathways. In the microbe-metabolites interaction analysis, we identified certain fatty acids (Dodecanedioic acid, Arachidic Acid, Mevalonic acid, etc.) that were significantly correlated with the MHO-enriched or depleted species.

**Conclusion:**

This study provides insights into identifying specific microbes and metabolites that may involve in the development of obesity without metabolic disorders. Future modalities for MHO intervention may be further validated by targeting these bacteria and metabolites.

## Introduction

The global prevalence of obesity has increased substantially, which continues to be an alarming public health issue (Blüher, 2019). Obesity is a complex condition that may contribute to impaired quality of life due to the development of associated disorders, including type 2 diabetes, cardiovascular diseases, and different types of cancers (Piché et al., 2020). However, the risks of developing such diseases vary widely among obese individuals even those with similar body mass index (BMI) (Neeland et al., 2019). Several clinical cohort studies have shown a subgroup of obese individuals with a lower risk of developing obesity-related cardiometabolic diseases (Cho et al., 2019; Fauchier et al., 2021). This phenotype is known as metabolically healthy obesity (MHO).

Although there is no universal definition, MHO is generally characterized by a BMI ≥30 kg/m^2^ without overt cardiometabolic syndromes at the time of diagnosis (Blüher, 2020). The prevalence of MHO has been reported to be up to 60% in all obese entities (Smith et al., 2019). Whereas there are decreased risks of cardiometabolic disorders in MHO individuals compared to those of unhealthy obese individuals, the risk is still higher in MHO groups compared to healthy lean individuals (Eckel et al., 2018). Thus, it is significant to identify the characteristics of MHO as a unique phenotype that may provide potential mechanisms linking risk factors promoting healthy weight gain to subsequent obesity-associated cardiometabolic complications.

Based on previous evidence, the gut microbiota is reported to be involved in obesity onset, progression, and complication development (Gupta et al., 2020). Distinct microbiome profiles exist between obese and non-obese individuals, such as the changed Firmicutes-to-Bacteroidetes ratio at the phylum level and the enrichment or depletion of various bacterial genera as reported by different studies (Kocełak et al., 2013; Kasai et al., 2015). However, most relevant research investigated mainly the intestinal microbiome profiles in unhealthy obesity patients compared with non-obese healthy controls. Few studies have specifically focused on the gut microbiota in the MHO phenotype. Two recent studies compared gut microbial composition between metabolically healthy and unhealthy obese individuals, but they only used the 16S rRNA sequencing data, which is limited to identifying significant microbial flora at the genus level (Kim et al., 2020; Zhong et al., 2020). Metagenomic studies are still lacking that independently examine the gut microbial difference at the species level between MHO groups and healthy lean individuals.

Here, we designed a paired-sample metagenomic analysis to identify promising microbial biomarkers contributing to MHO individuals. We independently compared differences in microbial profiles between MHO and healthy non-obese individuals by analyzing the data from an online human microbiome database. We also designed an *in vivo* study to detect the alteration of gut microbiota in the MHO phenotype, using the diet-induced obesity (DIO) mouse model. The present study can thus establish a more precise microorganism panel for the MHO phenotype.

## Methods

The present study includes two parts. First, we applied the online database (GMrepo, https://gmrepo.humangut.info) (Wu et al., 2020) to identify the gut microbial network and marker taxa from human individuals with MHO and healthy non-obese controls. Second, we investigated the differences in the compositions and functions of intestinal microbes/metabolites in the MHO mice model using metagenomic and metabolomics data.

### Study design and data collection from the GMrepo database

This study initially enrolled individuals in the GMrepo database with the health phenotype (Medical Subject Headings (MeSH) Unique ID: D006262). We classified eligible samples into two groups: MHO (BMI ≥30 kg/m^2^) and healthy non-obese controls (17<BMI<25 kg/m^2^).

### The matching process

First, projects containing the health phenotype were searched and identified. Unqualified Projects were excluded using the following criteria: (1) missing information on BMI, age, sex, or region, (2) negative quality control (QC) status, (3) unavailable microbial sequence data, and (4) failed runs of this phenotype. Next, samples within projects were categorized into the MHO and health groups. In each project, samples in MHO groups were matched with a 1:1 ratio to non-obese healthy controls. The matching criteria were age (+/- 3 years), sex (complete matching), region (complete matching), and Project ID (complete matching), using the module of the case-control matching in SPSS Statistics for Windows, Version 25.0 (IBM Corporation, Armonk, NY). Successfully matched samples in each project were merged and integrated for further analysis. The detailed procedure of data process and quality control in the database were described in Supplementary materials.

The data of relative abundance in each sample was collected and integrated into a microbial abundance table. The National Center for Biotechnology Information (NCBI) taxonomy database was used to classify organisms at different levels (Kingdom, Phylum, Class, Order, Family, Genus, Species). The taxonomic composition of each sample was then integrated into a final taxonomy classification table. Inclusion criteria included: (1) people with a healthy phenotype; (2) aged more than 18 years; (3) with positive QC status; and (4) with accessible metagenomes sequence data. Exclusion criteria were as follows: (1) a recent history of antibiotic use; (2) a sample with missing information on sex, age, region, or BMI; and (3) amplicon data only containing the abundance of bacteria at the genus level.

### Statistical analysis of the metagenomes data on the database

The diversities of the microbiome in the human data were evaluated by R software (version 4.1.0, http://www.R-project.org/). Alpha diversity was calculated by Richness (observed species) and Shannon and Simpson indices, using the vegan package in R. The differences in alpha diversity were calculated by the Wilcoxon signed rank test with continuity correction (“limma” package in R). Beta-diversity was presented by unconstrained principal coordinate analysis (PCoA) scatter plots by calculating Bray-Curtis distances. Permutational multivariate analysis of variance (PERMANOVA) was then used to determine the difference between different phenotypes (Kelly et al., 2015). The differential analysis of the microbial compositions in the MHO and control groups was evaluated using the packages “DeSeq2”, “edgeR”, and “Limma”. Clustering analysis with Log10 standardized data was applied to visualize the component difference of microbial species in the MHO and control groups, using the pheatmap package in R. The plot of clustering analysis, Venn, and species distribution in different subgroups were completed using the package “ggvenn” for Venn and package “ggplot2”, “tidyverse” and “reshape2” for species distribution plots. The Spearman correlation test was used to estimate the correlation between environmental factors and gut microbiota. Additionally, the randomForest package was applied to identify age/BMI-discriminatory bacterial taxa lists (Subramanian et al., 2014; Lou et al., 2021). The relative abundance of species was then regressed using default parameters, and the 20 most abundant species were used to map the developmental spectrum of gut microbiota in the MHO groups. Linear discriminant analysis effect size (LEfSe) was calculated with the online website (http://huttenhower.sph.harvard.edu/galaxy) to determine significant biomarkers for differentiating MHO and control samples. The cutoff value was defined if the linear discriminant analysis (LDA) score was more than 2.0 and P<0.05. Differences between groups were tested by the unpaired Mann–Whitney U test. Finally, a random forest model (Robin et al., 2011) was built. Significant microorganisms were incorporated into a panel for classifying MHO.

### Animal experiments

The animal study was performed following national legislation and was approved by the Institutional Animal Committee at the Laboratory Animals Center in Nanjing Medical University (2ACUC-2011028). Wild-type C57BL/6J mice (male, five weeks old) were purchased from the Beijing Vital River Laboratory Animal Technology Co., Ltd. (Beijing, China). After 1 week of adaptation, the mice were randomly assigned to 2 groups, including 20 mice fed on a high-fat diet (HFD: 60% kcal from fat; D12492, Research Diets), and 10 with a chow diet for eight weeks. At the beginning of the 9th week, the intraperitoneal glucose tolerance test (IPGTT) test was performed on all mice after 12 hours of overnight fasting. Serum levels of triglycerides (TG), cholesterol (TC), low-density lipoprotein cholesterol (LDL), and high-density lipoprotein cholesterol (HDL) were analyzed using a commercial assay kit (Nanjing Jiancheng Biotechnology Company, Nanjing, China).

### Fecal sample collection

Fresh fecal samples (2-3 pellets) were collected when the mouse defecated directly: 1) by first holding the mouse in one hand; 2) by transferring the sample with a disposable sterile plastic surgical tweezer (Cangzhou Hengbo Co., Ltd., Hebei, China) into a 2-ml sterile cryogenic vial (Corning Incorporated, USA); 3) quickly transferring the tube into a sealed sterile plastic bag and then storing it in a −80°C environment until further processing.

### Metagenomic analysis

DNA isolation was performed using the cetyltrim-ethylammonium bromide (CTAB). The quality control steps of DNA concentration and purification were performed using a Qubit^®^ dsDNA Assay Kit in Qubit^®^ 2.0 Fluorometer (Life Technologies, CA, USA). The qualified DNA sample was fragmented by sonication to a size of 350 bp. Library construction of the sequence was generated using the NEB Next^®^Ultra™ DNA Library Prep Kit for Illumina (Illumina, San Diego, CA, USA). Polymerase chain reaction (PCR) products were purified (AMPure XP system), and libraries were analyzed for size distribution by Agilent2100 Bioanalyzer (Agilent, Santa Clara, CA, USA) and quantified using real-time PCR. The clustering generation step of the index-coded samples was performed on a cBot Cluster Generation System, the library preparations were sequenced on an Illumina Novaseq 6000 platform, and paired-end reads were generated. The raw data were analyzed by Microeco Technology Co., Ltd. (Shenzhen, China). Cutadapt, KneadData, and FastQC were used for quality control of the raw data (based on Trimmomatic), and Kraken2 and the self-built microbial database were used for species annotation. Finally, a random forest model was built and significant microorganisms were incorporated into a panel for sorting MHO from healthy non-obese controls. All metagenomic raw data have been submitted to the Sequence Read Archive (SRA) in NCBI (number PRJNA853153).

### Metabolomic analysis

Fecal samples from mice were used for metabolomic analysis based on liquid chromatography/mass spectrometry (LC/MS) (Microeco Technology Co. Ltd. Shenzhen, China). Metabolite extraction was performed on a 100-mg fecal sample that was individually ground with liquid nitrogen (Mario et al., 2016). Ultraperformance liquid chromatography-tandem mass spectrometry (UHPLC-MS/MS) analyses were performed using a Vanquish UHPLC System (ThermoFisher, Germany) combined with an Orbitrap Q Exactive TM HF mass spectrometer (Thermo Fisher, Karlsruhe, Germany) by Novogene Co., Ltd. (Beijing, China). Samples were transferred into a Hypesil Gold column (100×2.1 mm, 1.9μm) using a 17-min linear gradient with a 0.2mL/min flow rate. Formic acid (0.1%) and ammonium acetate (5mM) were used as the solvent A for positive polarity mode and negative polarity mode, respectively. The solvent gradient B was set as follows: 2% B, 1.5 min; 2-100% B, 3 min; 100% B, 10 min; 100-2% B, 10.1min; 2% B, 12 min. The Q Exactive TM HF mass spectrometer was operated in +/- polarity mode with a 3.5 kV voltage, 320°C capillary temperature, 35 psi sheath gas flow rate, 10 L/min aux gas flow rate, 60 S-lens RF level, 350°C Aux Gas Heater temperature.

Raw data were then processed using Compound Discoverer 3.1 (CD3.1, Thermo Fisher) to perform peak alignment, peak picking, and quantitation for each metabolite. Peak intensities were normalized further to predict the molecular formula based on additive ions, molecular ion peaks, and fragment ions. Next, peaks were matched with the McCloud (https://www.mzcloud.org/), mzVaultand MassList database. Statistical analyses were performed using R software (version 3.4.3) and Python (version 2.7.6). Identified metabolites were annotated using the Kyoto Encyclopedia of Genes and Genomes (KEGG) database (https://www.genome.jp/kegg/pathway.html), Human Metabolome Database (HMDB) (https://hmdb.ca/metabolites), and LIPIDMaps database (http://www.lipidmaps.org/).

### Data analysis

The generated metagenomic data of mice were further analyzed using the MicrobiomeAnalyst (https://www.microbiomeanalyst.ca/) (Dhariwal et al., 2017), and metabolomic data were analyzed using MetaboAnalyst (https://www.metaboanalyst.ca/MetaboAnalyst/) (Pang et al., 2022). Molecular ecological network analyses (MENAs) were applied to construct random matrix theory (RMT) based on co-occurrence bacterial networks using Spearman’s correlation coefficient and presented in Grephi Version 0.9.5. The prediction of metagenome functions was visualized using Statistical Analysis of Metagenomic Profiles (STAMP) software (Version.2.1.3) (Parks et al., 2014).

Statistical analysis was performed using SPSS Statistics for Windows, Version 25.0 (IBM Corporation, Armonk, NY, USA). Normality tests were applied by Shapiro-Wilk and Kolmogorov-Smirnov tests. Data with a normal distribution were considered to have a *p*-value larger than 0.05 and are presented as the mean ± standard deviation, and data with a non-normal distribution are presented as median with interquartile range (Q). The Wilcoxon Mann-Whitney U tests were performed for unpaired data with a non-normal distribution. Pearson chi-square test was used to compare categorical variables with no more than 20% of cells with expected frequencies < 5, and Fisher’s exact test was used if > 20% of expected cell counts are less than 5 (Kim, 2017). Mantel analysis was performed to investigate the relationship of significantly different microbes/metabolites with clinical characteristics of mice, using the R packages of LinkET (Sunagawa et al., 2015).

## Results

### The baseline characteristics before and after the matching process

By searching the projects containing the health phenotype, we initially identified 130 projects in the database. Following the criteria, 107 projects were excluded for missing the data of BMI, age, sex, or region. Within the 23 projects, seven were excluded for negative QC status. A total of 16 projects followed the matching procedures. Four projects (PRJEB1220, PRJEB11419, PRJEB6997, PRJEB10878) were finally enrolled for further analysis, and fourteen were excluded without successfully matched pairs (**Table S1**). Within the four projects, 314 paired metagenomic samples (157 patients with MHO and 157 healthy non-obese controls were qualified for further analysis. The baseline information of the matched samples is presented in **Table 1** and **Table S2**.

**Table 1:** Baseline characteristics of patients in the GMrepo database.

Variable	*Before Match*			*After Match*		
	MHO (n=430)	Control (n=1028)	*p-value*	MHO (n=157)	Control (n=157)	*p-value*
Age (years, M, IQR)^†^	54 (10)	47 (26)	<0.001*	54 (12)	55 (13)	*0.416*
BMI (kg/m2, M, IQR)^†^	32.6 (4.34)	22.4 (2.78)	<0.001*	32.50 (5.13)	22.50 (2.19)	<0.001*
Country (n, %)			<0.001*			*1.000*
Denmark	347 (80.7%)	109 (10.6%)		79 (50.3%)	79 (50.3%)	
United States	59 (13.7%)	549 (53.4%)		58 (37.0%)	58 (37.0%)	
UK	15 (3.5%)	192 (18.7%)		14 (8.9%)	14 (8.9%)	
China	6 (1.4%)	102 (9.9%)		6 (3.8%)	6 (3.8%)	
Others	3 (0.7%)	76 (7.4%)		–	–	
Sex (n, %)			*0.075*			*1.000*
Male	237 (55.1%)	513 (49.9%)		69 (43.9%)	69 (43.9%)	
Female	193 (44.9%)	515 (50.1%)		88 (56.1%)	88 (56.1%)	
Age (n, %)			*0.788*			*0.316*
≥60 years	100 (23.3%)	246 (23.9%)		72 (45.9%)	96 (61.1%)	
<60 years	330 (76.7%)	782 (76.1%)		85 (54.1%)	61 (38.9%)	

p value was derived from the Mann-Whitney test in data of continuous variables with abnormal distribution (M, Median; IQR, Interquartile Range). The p value was derived from the chi-square test or Fisher’s exact test in data of categorical variables from MHO and healthy controls (n,%). MHO, metabolically healthy obesity; BMI, body weight index. †represents data of continuous variables with abnormal distribution, and * representssignificant p-values (<0.05).

### Alterations in the gut microbiota composition in MHO individuals

**Figure 1:**
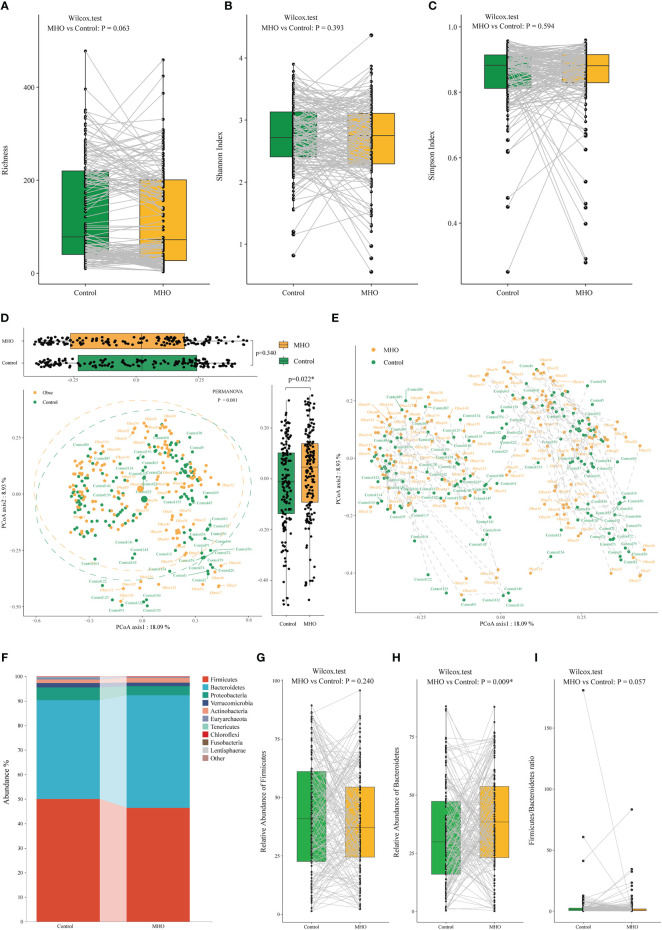
Alteration of gut microbiota in MHO individuals compared with their matched non-obese healthy controls according to the human metagenomic data. Alpha diversity was evaluated by the observed Richness **(A)**, Shannon index **(B)**, and Simpson index **(C)**. The gray lines indicate samples from MHO individuals and their matched non-obese healthy controls by age, sex, country, and Project ID. PCoAs of β-diversity were evaluated on species by Bray-Curtis distances **(D)**. Visualization of the distance of paired samples was shown in **(E)**. Each dot represents a sample. The difference in Bray-Curtis distances was calculated by the Mann–Whitney U test on Axis 1 (*p=*0.340) and 2 (*p=*0.022*), respectively. The dashed lines indicate a certain MHO patient was matched with a non-obese healthy control. The difference was calculated using the Wilcoxon Mann-Whitney U test. **(F)** The distribution of gut microbiota at the phylum levels in the both MHO and control groups. The abundance of Firmicutes **(G)**, Bacteroidetes **(H)**, and the F/B ratio **(I)** were compared between the two groups. MHO: Metabolically healthy obesity; PCoAs: Principal coordinate analyses.

A total of 2,487 microbial species were identified in both MHO and healthy non-obese groups. The richness of species, Shannon, and Simpson index in the MHO groups presented no difference when compared with controls (**Figures 1A–C**, **Table S3**). As for the beta diversity, we observed no difference in the Bray-Curtis distances in the Axis1 (*p=*0.340), but a significant difference in Bray-Curtis distances was present in the Axis2 (*p=*0.022) (**Figure 1D**). The PERMANOVA test also revealed a significant dispersion difference (*p<*0.001) between the MHO and controls. **Figure 1E** further visualized the distance in each paired sample.

At the phylum level, the MHO group was characterized by significantly higher *Bacteroidetes* levels (*p*=0.009), whereas the *Firmicutes/Bacteroidetes* ratio showed no difference in paired samples (*p*=0.057) (**Figures 1F–I**).

**Figure 2:**
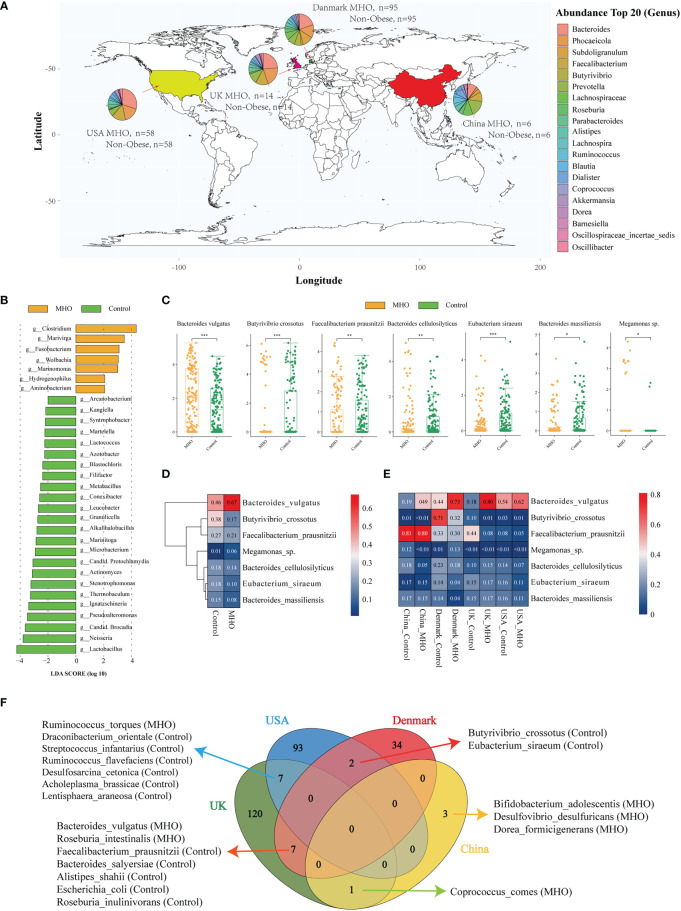
Significantly-changed species in MHO individuals. **(A)** Geographic distribution of samples from four different projects. Each pie chart represents the composition of the top 20 abundant genera in MHO subjects. **(B)** LEfSe analysis: Plot of LDA effect size. The length of the bar column represents the LDA score. The figure shows the microbial taxa with significant differences at genus levels between the MHO (Yellow) and Control (green) (LDA score>2.0). **(C)** The difference of relative abundance in certain species between MHO and controls (Log2 transformed). * p 0.05 ** p 0.01 *** p 0.001. **(D, E)** Heatmap visualization of the mean abundance in MHO patients and healthy controls based on regional differences. Each column represents one subgroup based on the MHO or control groups in different countries. Each row represents one of the seven species. Values in each square represent the mean relative abundance in percentage (Log 10 transformed). The color scale was set based on the specific value of the mean relative abundance after the Log 10 transformation, with red for relative high abundance and blue for the low ones. **(F)** Venn diagrams illustrating the number of significantly enriched or depleted species between MHO and non-obese healthy controls in different countries. The MHO or control in parentheses means the group in which the species was enriched.

The distribution of MHO genera profiles varies in different countries (**Figure 2A**). Within all samples, 481 species exclusively existed in the MHO group, and 519 were in the control groups (**Figure S1A**). In these 481 species, we noticed that *Streptococcus sobrinus* existed in MHO samples in three countries (**Figures S1B, C**). LEfSe analysis showed an overt alteration of the microbiota characterized by enriched genera of *Clostridium, Marivirga*, and *Fusobacterium* in MHO individuals with a linear discriminant analysis (LDA) >3. The abundance in the genera of *Lactobacillus, Neisseria, candidatus Brocadia, Pseudoalteromonas, Ignatzschineria, Thermobaculum, Stenotrophomonas*, and *Actinomyces* were higher in non-obese groups (**Figure 2B**, **Figure S1D**). Univariate analysis identified the five significantly different genera, including *Phocaeicola* (enriched in MHO, Wilcoxon rank-sum test, false discovery rate (FDR) <0.001), *Butyrivibrio* (depleted in MHO, FDR<0.001), *Barnesiella* (depleted in MHO, FDR<0.001), *Oscillospiraceae incertae sedis* (depleted in MHO, FDR<0.001), and *Paraprevotella* (depleted in MHO, FDR=0.009) (**Figure S1E**, **Table S4**).

At the species level, species of *Bacteroides vulgatus* (*B.vulgatus*) (Wilcoxon rank-sum test, FDR<0.001) and *Megamonas* sp. (FDR=0.042) were significantly enriched in MHO groups, whereas *Butyrivibrio crossotus* (*B.crossotus*) (FDR<0.001), *Faecalibacterium prausnitzii* (*F.prausnitzii*) (FDR=0.007), *Bacteroides cellulosilyticus* (*B.cellulosilyticus*) (FDR=0.005), *Eubacterium siraeum* (*E.siraeum*) (FDR<0.001), and *Bacteroides massiliensis* (FDR=0.024) were significantly enriched in non-obese controls (**Figure 2C**, **Table S5**). A random forest model was established to identify the top 50 important biomarkers to differentiate MHO from non-obese controls. *F.prausnitzii, B.crossotus*, and *B.vulgatu*s were the top 3 most important microbial markers for differentiating MHO from health. (**Figure S1F**)

As geographical location may exhibit a great influence on the gut microbiota, we stratified data into subgroups by different regions. **Figures 2D, E** shows the expression of the seven significantly changed species in different countries. *B.vulgatus* and *F.prausnitzii* showed the same pattern of abundance change in all sub-regions, but the patterns of expression on the other 5 species are not consistent among regions. Independent differential microbiome analyses were then performed in each sub-region group. Significantly-changed species in each sub-regional group were shown in **Table S6**. Among significantly-different species, *B.crossotus* and *E.siraeum* were commonly identified in USA and Denmark samples as significantly depleted in MHO groups. *B.vulgatus* and *F.prausnitzii* and the other five species were commonly shown in Denmark and UK subgroups as significantly changed in MHO groups. In UK and USA cohorts, *Ruminococcus torques* were significantly enriched in MHO groups, whereas the other six species were significantly depleted (**Figure 2F**).

### Relationship of gut microbiota and age or BMI

In all samples, the abundance of *B.vulgatus* (Spearman correlation test, r=0.20, FDR=0.001) was positively correlated to the elevated BMI, whereas *B.cellulosilyticus* (r=-0.21, FDR<0.001) and *E.siraeum* (r=-0.22, FDR<0.001) had negative correlations with BMI increase. As for the relationship with age, increased enrichment of *Veillonella* sp. (r=0.23, FDR<0.001) and *R.torques* (r=0.19, FDR=0.002, *Subdoligranulum* sp. (r=0.22, FDR<0.001), and *Megamonas* sp. (r=0.17, FDR=0.006) were significantly correlated with increased age (**Figure S2**, **Table S7**). We further tracked the principal microbial strains associated with age and BMI in MHO individuals using random forest algorithms. **Table S8** listed the top 20 significant species with age- and BMI-mediated changes in abundance. **Supplementary Figure S3** presents the scatter plots of the highly significantly correlated microbes with age (S3A) and BMI (S3B). We found a decreasing trend in *B.vulgatus* which was correlated with increasing age. *Bacteroides thetaiotaomicron* (*B.thetaiotaomicron*) and *R.torques* were enriched in elderly MHO patients (age>60 years old). *Parabacteroides distasonis* (*P.distasonis*) showed a decreasing trend when BMI exceeded 30 kg/m2.

### Taxonomic composition of the gut microbiota in the MHO murine model

**Figure 3:**
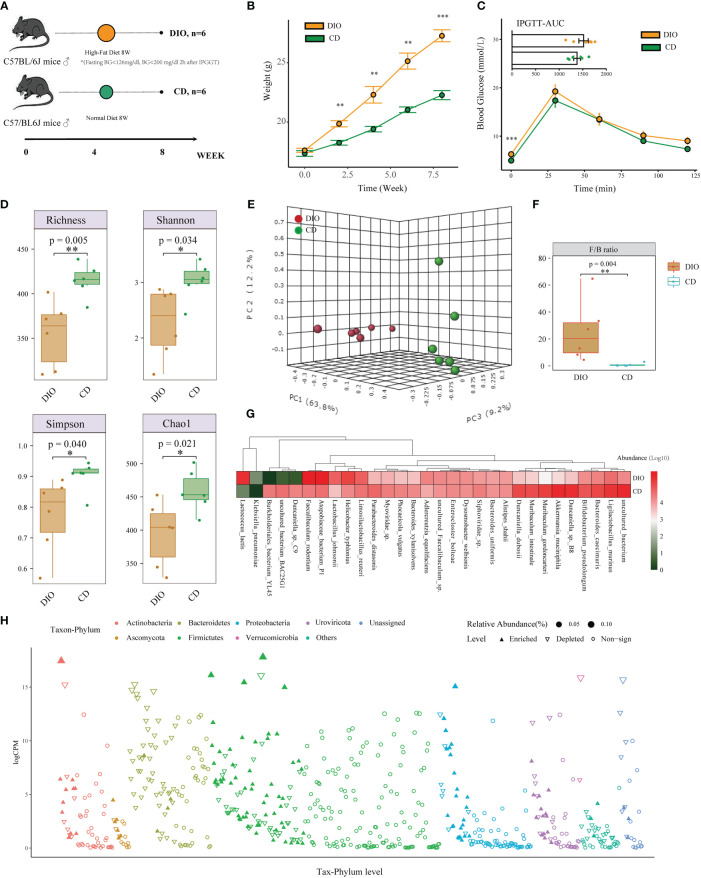
Alteration of Taxonomic Composition in MHO Mice. **(A)** Experimental design of mice. **(B)** Metabolic parameters of body weight **(B)** and IPGTT **(C)** were examined at the beginning of the 9th week. **(D)** Alpha diversity was evaluated by observed Richness, Shannon index, Simpson index, and Chao1 using the Student’s *t*-test. **(E)** PCoAs of β-diversity with the 3-dimensional presentation. **(F)** Firmicutes/Bacteroidetes ratio was compared using the Mann-Whitney U test between MHO and controls. **(G)** Heatmap visualization of the top 30 abundant species in MHO and controls. **(H)** Manhattan plots showing the changed composition of species in MHO by the edgeR algorithm. The color of each dot represents the different taxonomic affiliations of the species (phylum level), and the size corresponds to their relative abundance. The Y-axis represents log counts per million(CPM). The upward-pointing triangle indicates enriched species, whereas the downward-pointing triangle represents depleted ones. * p 0.05 ** p 0.01 *** p 0.001.

Besides the analysis in human projects, we also designed a mice study to detect the changes in composition and function of the intestinal microbiome in MHO phenotype, using the metagenomic and metabolomic analysis from mice fecal samples (**Figure 3A**). The MHO mice model in this study is defined as fatty mice (HFD for 8 weeks) with a fasting BG<7.0mmol/L (126mg/dl) and BG<11.0mmol/L (200 mg/dl) 120 min after IPGGT. Among all 20 HFD mice, 6 were finally selected as qualified MHO mice. For comparison, we randomly selected 6 of 10 lean mice with a chow diet as the final controls. **Figure 3B** presents the weight change during the eight-week experiment (**Table S9**). After eight weeks of HFD, the mean weight was significantly higher (*t*-test 22.27 v.s. 27.27 g, *p*<0.001) in DIO mice than in controls (**Figure 3B**). No difference in IPGTT-AUC (1516.25 v.s. 1378.75 mmol/L*min, *p=*0.289) was observed in the two groups (**Figure 3C**, **Table S10**). The serum concentrations of TG, TC, and LDL were significantly higher in MHO groups, whereas the HDL concentration showed no difference in both groups (**Figure S4A**).

A Read summary of the metagenomic data is shown in **Table S11**. A total of 966 species were identified in both groups. Significant differences were observed in the number of observed species (*p=*0.005), the Simpson’s index (*p=*0.040), Shannon’s index (*p=*0.034), and the Chao1 index (*p=*0.021) using the Student’s *t*-test (**Figure 3D**). The beta diversity (PERMANOVA, R: 0.60872; *p*<0.003) revealed a significant difference in the gut microbiota between the MHO and controls (**Figure 3E**). The abundance of species belonging to *Bacteroidetes* was depleted in MHO, whereas that belonging to *Firmicutes* was enriched. The *Firmicutes/Bacteroidetes* ratio was significantly increased in the MHO group (**Figure 3F**, **Figure S4B**).

**Figure 3G** shows the relative abundance of the top 30 species in the two groups using a heat-map plot. Different compositions of certain species were identified by using five different algorithms (MetagenomeSeq, EdgeR, DESeq2, LEfSe, and Wilcoxon Test). The EdgeR results are visualized in **Figure 3H**. Species with significantly different abundance in the two groups belonged mainly to the phyla *Firmicutes* and *Bacteroidetes*.

**Figure 4:**
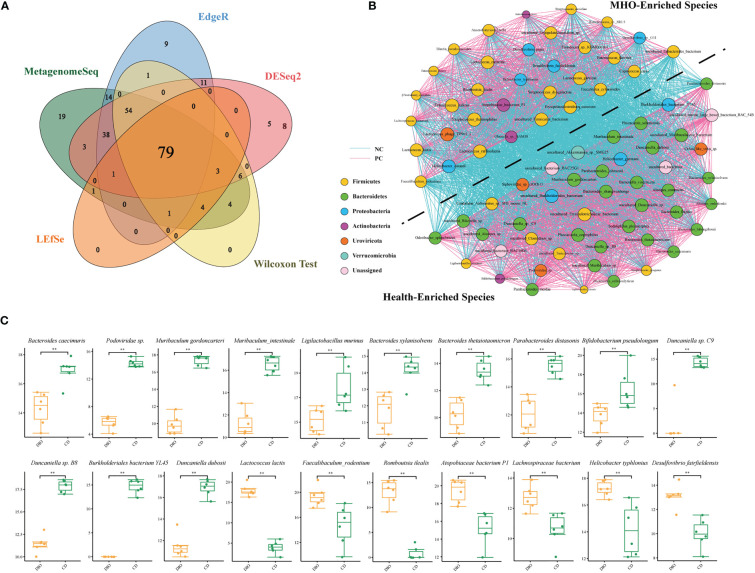
Significantly-changed species in MHO mice. **(A) **Venn diagrams illustrating 79 species commonly identified using five different algorithms (MetagenomeSeq, EdgeR, DESeq2, LEfSe, and Wilcoxon Test). **(B)** Co-occurrence network visualization of the interactions among the above species. The lines connecting nodes (edges) represent a positive (light blue) or negative (pink) co-occurrence relationship. The color of each dot represents the different taxonomic affiliations of the species (phylum level), and the size corresponds to their relative abundance. **(C)** For some representative species among the above 79 species, the difference in abundance was compared using the Mann-Whitney U test. * p 0.05 ** p 0.01.

Next, we used a Venn diagram and found 79 common bacteria identified as significantly-different species in the two groups using the above five algorithms (**Figure 4A**, **Table S12**). A cooccurrence network was generated among these remarkably changed species using the Spearman analysis (**Figure 4B**, **Table S13**). Species belonging to the *Bacteroidetes* phylum are depleted mainly in MHO mice, whereas species belonging to the *Firmicutes* phylum are enriched in MHO mice. There is an overt negative-correlated relationship between obesity-enriched species and nonobese-enriched species. Of the 79 significantly-changed bacteria, 20 were within the top 50 species ranked by relative abundance in both MHO and control groups (**Figure 4C**). Among these bacteria, thirteen species were significantly enriched, whereas seven were significantly depleted in the MHO groups.

### Alteration of bacteria functions in MHO mice

**Figure 5:**
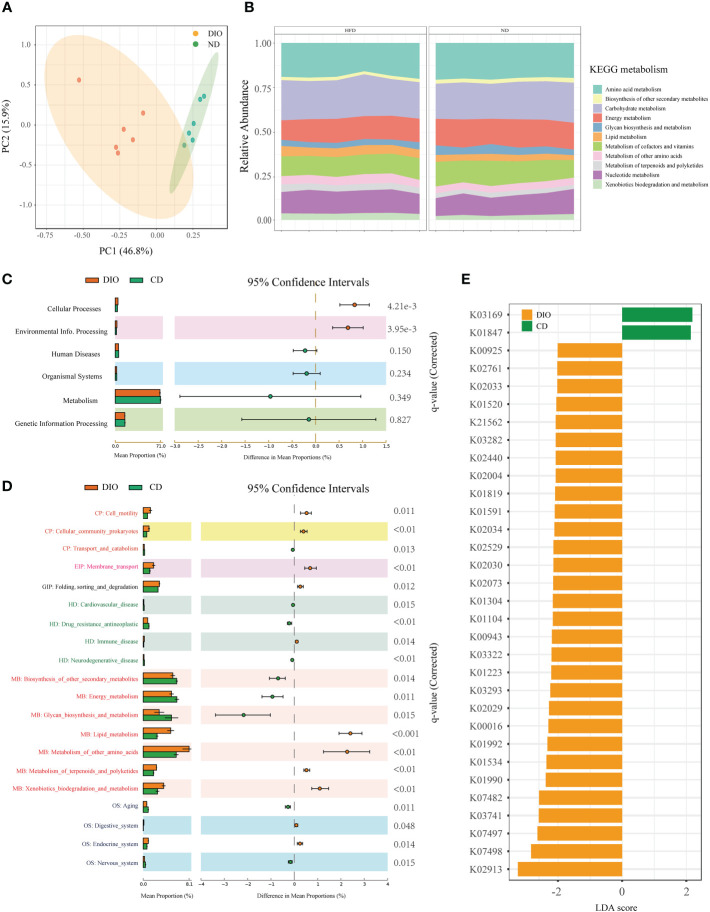
Microbial gene function annotation by KEGG in MHO and controls. **(A)** PCoA based on KEGG modules revealed differences in microbial functions between DIO and controls. **(B)** The composition of KEGG modules in both MHO and controls. The KEGG pathways of level 1 **(C)**, level 2 **(D)**, and KO **(E)** were all disrupted in the MHO groups, the relative to healthy controls, as identified by STAMP software.

Functional analysis for metagenomic data were shown in **Figure 5**. PCoA based on KEGG modules revealed differences in microbial functions between MHO and controls (**Figure 5A**). **Figure 5B** shows the compositions of KEGG modules in both MHO and control groups. Meanwhile, the KEGG pathways of level 1, level 2, and KEGG Orthology (KO) were all disrupted in the MHO groups, relative to the healthy controls (**Figures 5C** **–E**). The metabolic pathway activity of energy was lower, whereas the lipid and amino acids pathways were higher in the MHO groups.

### Metabolomics analysis revealed aberrant metabolic patterns in MHO mice

**Figure 6:**
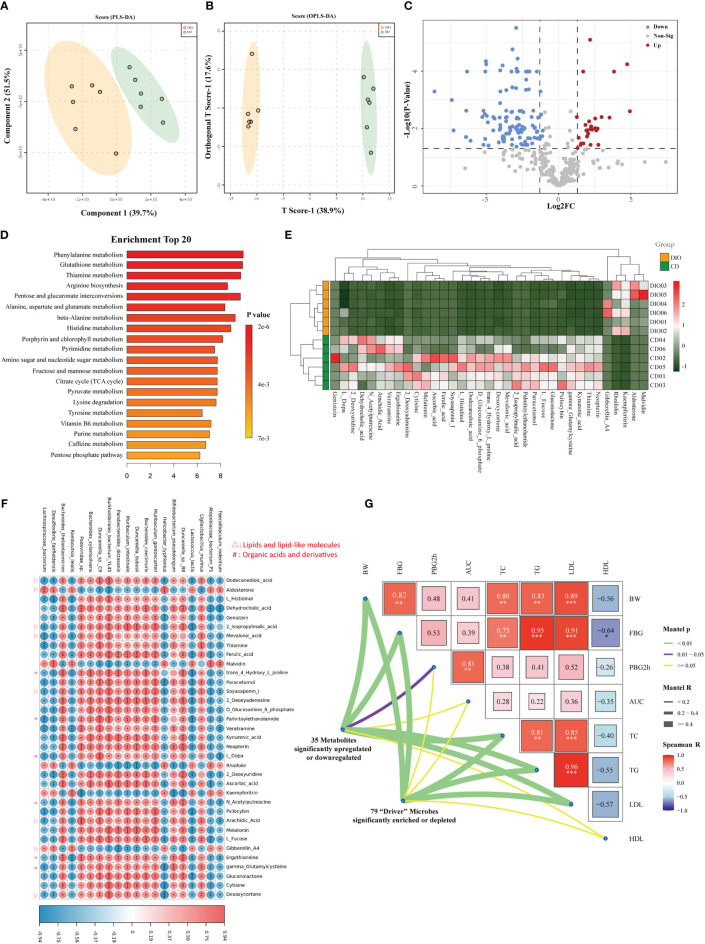
Metabolomics analysis revealed aberrant metabolic patterns in MHO mice. Both PLS-DA **(A)** and OPLS-DA **(B)** showed a significantly-different distribution of metabolites in MHO and controls. **(C)** Volcano plot. The log2 fold-change indicates the mean relative abundance for each metabolite. Each dot represents one metabolite. The gray dots represent no significant expression difference between the MHO and control groups, the red dots represent MHO-enriched metabolites, and the blue dots represent MHO-depleted metabolites. **(D)** Enrichment analysis of differentially expressed metabolites shows the top 20 enriched pathways in MHO metabolism. **(E)** Heatmap visualization of the thirty-five metabolites with |LogFC|>4 in the MHO groups compared with the controls. **(F)** A correlation heatmap linking the top 20 significantly-differentiated bacteria with the above 35 significantly-changed metabolites. **(G)** The triangle on the right side represents pairwise comparisons of clinically relevant factors with a color gradient denoting Spearman correlation coefficients. The 79 significantly enriched or depleted microbes and 35 significantly changed metabolites were related to each clinical factor, respectively, using partial (geographic distance– corrected) Mantel tests. Edge width corresponds to the Mantel r statistic for the corresponding distance correlations, and edge color denotes the statistical p significance value. PLS-DA: The Partial least squares discriminant analysis; OPLS-DA: Orthogonal Partial least squares discriminant analysis. * p 0.05 ** p 0.01 *** p 0.001.

**Figure 7:**
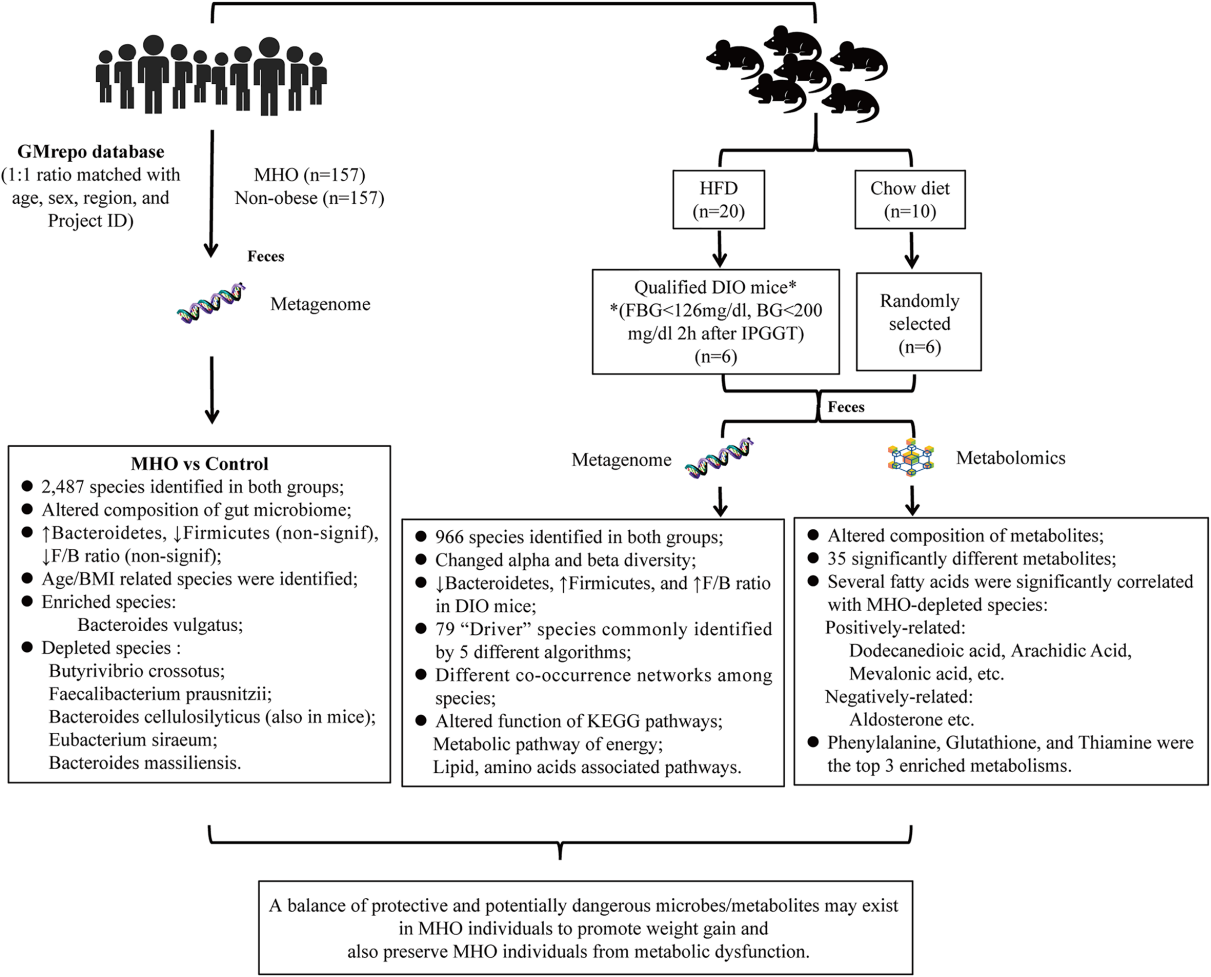
Summary of the study.

We explored the metabolic profile in the same samples from metagenomics analysis (six MHO mice and six lean controls). A total of 347 metabolites (with distinct IDs in the KEGG database and HMDB) were identified in both groups (**Table S14**). The fecal samples were obviously classified according to the partial least squares discriminant analysis (PLS-DA) and orthogonal partial least squares discriminant analysis (OPLS-DA) (**Figures 6A, B**), indicating different metabolic pathways between the two groups. A total of 128 metabolites were identified as significantly-different materials between the two groups (Log fold change (FC)>2, FDR<0.05) (**Figure 6C**, **Table S15**). Twenty-three KEGG pathways were significantly different between the two groups (FDR<0.05) (**Table S16**). **Figure 6D** presents the top 20 significantly-changed pathways in the MHO group. Enrichment analysis of metabonomic data showed that the MHO phenotype interrupts mainly energy intake and the metabolic pathway of lipids, amino acids, vitamins, etc. The top three enrichment pathways were the metabolism of phenylalanine, glutathione, and thiamine. Thirty-five metabolites with |Log FC|>4 were shown in **Figure 6E** and **Table S17**. These materials belong mainly to lipids or lipid-like molecules, organic compounds, acids, and derivatives (including amino acids). We performed a correlation matrix by linking the top 20 significantly-differentiated bacteria with the above 35 metabolites (**Figure 6F**, **Table S18**). Among the 35 metabolites, all fatty acids (Dodecanedioic acid, Arachidic Acid, Mevalonic acid, Soyasaponin I, 2-Isopropylmalic acid, and Desoxycortone) were positively correlated with MHO-depleted species while Aldosterone and Gibberellin A4 were negatively-related with MHO-enriched species. To verify the clinical independence of remarkable microbes/metabolites in MHO phenotype, we correlated some clinically relevant factors with distance-corrected dissimilarities of taxonomic and metabolic compositions. The body weight, fasting glucose levels, and serum levels of TC, TG, and LDL were strongly correlated with remarkably changed microbes and metabolites (**Figure 6G**). A summary of the study results is presented in **Figure 7**.

## Discussion

This is the first integrated multiomics analysis that independently investigated the interaction between the gut microbiota and the MHO phenotype. In the first part, we applied a paired-sample design using matched age, sex, and region among the same projects to reduce confounding biases. We highlighted several bacteria with remarkably different distributions between MHO and non-obese controls from human fecal data. *B.vulgatus* was a significantly-enriched species within the top 50 most abundant microbes in MHO individuals, and the abundance of *B.vulgatus* was increased in the MHO group in all countries within our study. *B. vulgatus* has been reported to possibly participate in preventing the pathogenesis of atherosclerosis by reducing lipopolysaccharide production in the gut (Yoshida et al., 2018). Thus, we assume that the enrichment of *B.vulgatus* may serve as a protective agent in MHO individuals to stay away from cardiometabolic syndromes. We also noticed that the abundance of *B.vulgatus* was negatively correlated with age, indicating that such protection may potentially decrease in elderly individuals. In contrast, *F.prausnitzii* and *B.crossotus* were two MHO-depleted species from our results. *F.prausnitzii* has been identified as a butyrate-producing bacterium, and it might function in the prevention and therapy of obesity-associated disease due to its production of anti-inflammatory metabolites (Hippe et al., 2016; Maioli et al., 2021). *B.crossotus* has been reported to have decreased abundance in metabolic disorders, and high levels of *B.crossotus* might protect against weight gain (Le Chatelier et al., 2013; Grigor’eva, 2020). In other words, both *F.prausnitzii* and *B.crossotus* are protective species against obesity. The depletion of these two microbes may promote the incidence of obesity in nonobese individuals.

Considering that diet and other environmental factors may interrupt the composition of gut microbiota in MHO and non-obese people, we applied mouse models for further comparison. The qualified MHO mice were set as diet-induced obese mice without overt impaired glucose tolerance. First, we observed a remarkable richness of species in human fecal samples compared with those in mice. Additionally, compared with nonobese groups, there was a more apparent decreasing trend of alpha diversity in MHO mice than in human samples. Besides, we noticed an opposite trend in humans and mice regarding the distribution of *Bacteroidetes* and *Firmicutes*. In human samples, we observed enriched *Bacteroidetes* and decreased *Firmicutes*/*Bacteriodetes* (F/B) ratio (without statistical significance) in the MHO group. In mouse samples, however, there were significantly depleted *Bacteroidetes*, enriched *Firmicutes*, and an increased F/B ratio in MHO mice. This inconsistent result was also shown in previous studies in which the F/B ratio varied among different obese cohorts (Jumpertz et al., 2011; Kocełak et al., 2013; Million et al., 2013). Thus, we suggest that the F/B ratio might not be used as a microbial biomarker to distinguish obesity from healthy individuals.

We identified 79 “driver” species in mice, commonly presented in five different algorithms, to distinguish MHO from non-obese controls. Among these driver species, we observed the enrichment of some protective bacteria in the MHO groups, such as *Lactococcus lactis*, (*L.Lactis*) *Clostridium cocleatum* (*C.cocleatum*), and *Enterococcus faecium* (*E.faecium*). *L.Lactis* is reported to protect against metabolic changes induced by Western diets (Naudin et al., 2020). The richness of *C.cocleatum* participates in metabolic improvement after metformin intake (Lee and Ko, 2014). *E.faecium* may prevent obesity-associated hyperlipidemia (Huang et al., 2021). These species may participate in maintaining the stability of the MHO phenotype by preventing obesity-related metabolic diseases. However, the abundance of some protective bacteria was decreased in MHO mice, including *B.cellulosilyticus, P.distasonis, B.thetaiotaomicron, and Muribaculum intestinale*, which may promote weight gain in mice. We noticed that *B.cellulosilyticus* was depleted in both the MHO groups of human and mouse fecal samples. It is reported that the enrichment of *B.cellulosilyticus* is associated with a healthy plant-based diet index, indicating a lower risk of cardiometabolic conditions (Li et al., 2021). Thus, the depletion of *B.cellulosilyticus* may potentially take part in obesity pathogenesis in nonobese individuals.

Next, we explored the remarkably-changed metabolites in the MHO phenotype. Phenylalanine, glutathione, and thiamine were the top 3 enriched metabolisms that have been reported to participate in the process of cardiometabolic complications of obesity (Fan et al., 2020; Arnoriaga-Rodríguez et al., 2020; Alkazemi et al., 2021). Among the 35 significantly-changed metabolites, we found changed activities of lipids molecules correlated with MHO phenotype. Dodecanedioic acid (DA), arachidic acid (AA), and mevalonic acid (MVA) are fatty acyls that may be involved in antiobesity activities, such as the inhibition of inflammation, prevention of weight gain, postponing muscle fatigue, and acceleration of fatty acid utilization (Salinari et al., 2006; Shao et al., 2021; Ahmad et al., 2022). These fatty acyls were decreased in MHO mice, indicating a potential risk of weight gain and may also be involved in metabolic syndromes. We further noticed that some anti-obesity species such as *P.distasonis*, *B.thetaiotaomicron*, and *Muribaculum intestinale* were strongly correlated with these fatty acyls, with the same decrease trend in MHO mice. Thus, the depletion of these microbes might diminish anti-obesity activities by reducing fatty acid utilization. Paradoxically, these depleted fatty acyls were also strongly correlated with the increasing growth of other potentially beneficial species, such as *L.lactis* and *Faecalibaculum rodentium*. Although all these bacteria had a potentially protective function, *P.distasonis*, *B.thetaiotaomicron*, and *Muribaculum intestinale* were negatively correlated with *L.lactis* and *Faecalibaculum rodentium* in the microbial interaction network. The possible explanation for this paradoxical phenomenon is that a potential balance of microbe interactions may exist in the gut environment. Although the depletion of some anti-obesity bacteria identified in MHO may promote weight gain in the healthy phenotype, it may also promote the growth of other obesity-protective bacteria, which might preserve MHO individuals from metabolic dysfunction.

Interestingly, such a balance of protective microbes/metabolites and potentially dangerous materials may exist in MHO individuals. On the one hand, some species/metabolites may be involved in the initial weight gain at nonobese status. On the other hand, other groups of protective species/metabolites may also preserve MHO from metabolic dysfunctions. We speculate that if the microbiota dysbiosis becomes more severe, such balance in the MHO may be further interrupted, and relevant species/metabolites might also be involved in the gradual shifts of MHO individuals toward morbid obesity. Thus, future studies should be performed to validate the function of these core species and their associated metabolites in preserving the stability of the MHO phenotype.

Our study has some limitations. First, we were limited to accessing detailed human data on clinical characteristics such as blood glucose, waist circumstance, blood pressure, TC, and TG levels, due to the unavailability of this information in the GMrepo database. Second, as the GMrepo database does not provide the ready-to-use functional profile, the KGEE analysis of the human metagenomic data was not performed in this study. In the sub-regional differential analysis, we used unadjusted p-values to identify significant species and this may statistically increase the false positive rate in that part. Furthermore, the sample size in the murine study is relatively small, and the microbe and metabolite interactions may not be fully interrupted, as the current fecal metabolism analysis cannot completely illustrate the natural metabolic state of the host. Thus, future studies targeting the associated analysis of the metabolism with fecal microbiota metabolites are still needed to further explore the metabolic role in the pathological mechanism of the MHO phenotype.

In summary, this study provides insights into identifying specific microbes and metabolites that may involve in the development of obesity without metabolic disorders. Possible future modalities for MHO intervention may be further validated by targeting these bacteria and metabolites.

## Data sharing

Metagenomic data of gut microbiota associated with “Metabolically healthy obesity” mice. BioProject Accession: PRJNA853153; ID: 853153.

## Data availability statement

The datasets presented in this study can be found in online repositories. The names of the repository/repositories and accession number(s) can be found in the article/**Supplementary Material**.

## Ethics statement

The animal study was performed following the national legislation and was approved by the Institutional Animal Committee at the Laboratory Animals Center in Nanjing Medical University (2ACUC-2011028).

## Author contributions

HanC: study concept and design, analysis and interpretation of data; drafting of the manuscript, authorship; NT: study concept and design, analysis and interpretation of data, authorship. QY: data extraction, design and order the figures and tables, assessment of study quality, authorship. XY, RY and HongC: design and order the figures and tables, assessment of study quality. GZ and XZ: critical revision of the manuscript for important intellectual content; obtain funding; study supervision, authorship. All authors contributed to the article and approved the submitted version.

## Funding

This research was supported by grants from the National Natural Science Foundation of China (82100594). The study has not received any funding or grants from pharmaceutical or other industrial corporations. 

## Conflict of interest

The authors declare that the research was conducted in the absence of any commercial or financial relationships that could be construed as a potential conflict of interest.

## Publisher’s note

All claims expressed in this article are solely those of the authors and do not necessarily represent those of their affiliated organizations, or those of the publisher, the editors and the reviewers. Any product that may be evaluated in this article, or claim that may be made by its manufacturer, is not guaranteed or endorsed by the publisher.

## Abbreviations

Metabolically healthy obesity (MHO); Diet-induced obesity (DIO); Liquid chromatography-mass spectrometry (LC/MS); Body mass index (BMI); Medical subject headings (MeSH); Quality control (QC); Principal coordinate analyses (PCoA); Permutational multivariate analysis of variance (PERMANOVA); Partial least squares discriminant analysis (PLS-DA); Orthogonal partial least squares discriminant analysis (OPLS-DA); Linear discriminant analysis effect size (LEfSe); Linear discriminant analysis (LDA); Specific-pathogen-free (SPF); Intraperitoneal glucose tolerance test (IPGTT); Cetyltrimethylammonium bromide (CTAB); Kyoto Encyclopedia of genes and genomes (KEGG); Random matrix theory (RMT); The molecular ecological network analyses (MENA); Statistical analysis of metagenomic profiles (STAMP); Dodecanedioic acid (DA); Arachidic acid (AA); Mevalonic acid (MVA); *Firmicutes/Bacteroidetes* ratio (F/B ratio).
