# Supplementary material for: Alternation of the gut microbiota in metabolically healthy obesity: An integrated multiomics analysis
Source: Front Cell Infect Microbiol. 2022 Nov 1;12:1012028. doi: 10.3389/fcimb.2022.1012028 (PMC9663839; doi:10.3389/fcimb.2022.1012028)
Supplement: Supplementary file 1 [file DataSheet_1.zip › Supplementary online materialV2.docx]

# Supplementary online material

*Data processing on the GMrepo database*

FastQC (version 0.11.8) was used to evaluate the overall quality of the downloaded data, followed by the use of Trimmomatic to remove vector sequences and low-quality bases. Sequences shorter than ⅔ of the original read length were removed from subsequent analysis. The remaining sequences were referred as to clean data and used for subsequent analysis.

*Quality control on the GMrepo database*

Two rounds of quality control procedures were applied. First, for the 16S data, samples (runs) with less than 20,000 clean reads were removed from the subsequent analysis and marked as "failed QC (QC status == 0)" in GMrepo. Then, after taxonomy assignment, samples containing only a single taxon (i.e., a species or genus that accounts for more than 99.99 percent of total abundance) will be marked as "failed QC (QC status == 0)".

*Taxonomic classification on the GMrepo database*

For whole genome (i.e., metagenomic or mNGS) sequences, MetaPhlAn2 was used with default parameters for the taxonomic classification of the sequencing reads.

*The KEGG levels of metabolites in the murine study*

| **KEGG Level1** | **KEGG Level2** |
| --- | --- |
| Cellular Processes | Cellular Processes;Cell growth and death |
|  | Cellular Processes;Cell motility |
|  | Cellular Processes;Cellular community _ eukaryotes |
|  | Cellular Processes;Cellular community _ prokaryotes |
|  | Cellular Processes;Transport and catabolism |
| Environmental Information Processing | Environmental Information Processing;Membrane transport |
|  | Environmental Information Processing;Signal transduction |
|  | Environmental Information Processing;Signaling molecules and interaction |
| Genetic Information Processing | Genetic Information Processing;Folding, sorting and degradation |
|  | Genetic Information Processing;Replication and repair |
|  | Genetic Information Processing;Transcription |
|  | Genetic Information Processing;Translation |
| Human Diseases | Human Diseases;Canceroverview |
|  | Human Diseases;Cancerspecific types |
|  | Human Diseases;Cardiovascular disease |
|  | Human Diseases;Drug resistanceantimicrobial |
|  | Human Diseases;Drug resistanceantineoplastic |
|  | Human Diseases;Endocrine and metabolic disease |
|  | Human Diseases;Immune disease |
|  | Human Diseases;Infectious diseasebacterial |
|  | Human Diseases;Infectious diseaseparasitic |
|  | Human Diseases;Infectious diseaseviral |
|  | Human Diseases;Neurodegenerative disease |
|  | Human Diseases;Substance dependence |
| Metabolism | Metabolism;Amino acid metabolism |
|  | Metabolism;Biosynthesis of other secondary metabolites |
|  | Metabolism;Carbohydrate metabolism |
|  | Metabolism;Chemical structure transformation maps |
|  | Metabolism;Energy metabolism |
|  | Metabolism;Global and overview maps |
|  | Metabolism;Glycan biosynthesis and metabolism |
|  | Metabolism;Lipid metabolism |
|  | Metabolism;Metabolism of cofactors and vitamins |
|  | Metabolism;Metabolism of other amino acids |
|  | Metabolism;Metabolism of terpenoids and polyketides |
|  | Metabolism;Nucleotide metabolism |
|  | Metabolism;Xenobiotics biodegradation and metabolism |
| Organismal Systems | Organismal Systems;Aging |
|  | Organismal Systems;Circulatory system |
|  | Organismal Systems;Development and regeneration |
|  | Organismal Systems;Digestive system |
|  | Organismal Systems;Endocrine system |
|  | Organismal Systems;Environmental adaptation |
|  | Organismal Systems;Excretory system |
|  | Organismal Systems;Immune system |
|  | Organismal Systems;Nervous system |
|  | Organismal Systems;Sensory system |

**Suppl. Figures**

**Figure S1. The ditribution of species in different countries.** (A) Venn diagrams illustrating the number of species between MHO (yellow) and healthy controls (green). (B) Venn diagrams based on subgroup of different countries. (C) UpSet plot of differently-distributed taxa. The left graph represents the total number of differently-distributed species (X-axis) in different countries (Y-axis). The right graph represents the intersection of sets of species in multiple countries. Each column corresponds to a country or set of countries (dots connected by lines below the X-axis) containing the same species. The number of species in each set appears above the column, while countries shared are indicated in the graphic below the column. (D) Cladogram Plot by LEfSe analysis. (E) Univariant analysis identified the five significantly different genera using Wilcoxon rank-sum test. (* p 0.05 ** p 0.01 *** p 0.001.) (F) A random forest model of the top 50 biomarkers to differentiate MHO from non-obese controls.

Figure S2. **Relationship of gut microbiota and age/BMI in MHO groups (Spearman).** A: The heatmap reporting correlation coefficients (Rho) and *p* values for correlation of species and age/BMI. The row represents certain species, and the column represents age and BMI. The bar on the right side shows the color scale reflecting the Rho values positive correlations of a species with age or BMI (Rho > 0) are presented as purple-scale squares, whereas negative correlations (Rho ＜ 0) as green squares. The more weight of the absolute Rho value, the deeper the color bar presents. "*" represents the p-value of the correlation. (The Spearman rank correlation test, *, **, *** stands for p-value < 0.01, 0.005 and 0.001, respectively). B: Scatter plots of highly significantly correlated microbes and factors of BMI and scatter plots of some significantly different species (*Faecalibacterium prausnitzii, Butyrivibrio crossotus, Bacteroides massiliensis, and Megamonas sp*) identified previously in the differential analysis. C: Scatter plots of highly significantly correlated species and factors of age (r>0.15 or *p*<0.001).

**Figure S3. Relationship of gut microbiota and age/BMI in MHO groups (Random Forest methods).** (A) The correlation heatmap represents significant statistical correlation values (Rho) between gut microbiota and clinical features of age and BMI. In heatmap, purple squares indicate significant positive correlations (Rho > 0.5, p < 0.05) and green squares indicate significant negative correlations (Rho < − 0.5, p < 0.05). *, **, *** represents p-value < 0.01, 0.005 and 0.001 respectively). (B-C) The alteration of relative abundance in the top 20 important species identified by random forest algorithm in MHO patients with constantly changed age and BMI.

**Figure S4.** (A) Boxplot of the serum concentrations of TG, TC, LDL, and HDL in the murine study. T-test was performed to compare the mean of each value between the DIO and control groups, and *, **, *** stands for p-value < 0.01, 0.005 and 0.001, respectively). (B) The distribution of Firmicutes and Bacteroidetes in mice feces samples.

**Suppl.Tables**

Table S1. The detailed information of identifying qualified projects in the GMrepo database.

Table S2. Baseline information of the human cohort in GMrepo database.

Table S3. Data of Alpha diversity in human samples.

Table S4. The top 50 abundant genera in MHO and control groups.

Table S5. The top 50 abundant species in MHO and control groups.

Table S6. Significantly-changed species in each sub-regional group.

Table S7.Relationship of gut microbiota and age/BMI in MHO groups (Spearman).

Table S8. Relationship of gut microbiota and age/BMI in MHO groups (Random Forest).

Table S9. Weight change of mice during the animal experiment.

Table S10. IPGTT results of mice during the animal experiment.

Table S11. Reads summary of metagenomic data in mice feces.

Table S12. The 79 species commonly identified using five different algorithms (MetagenomeSeq, EdgeR, DESeq2, LEfSe, and Wilcoxon Test).

Table S13. Spearman results of co-occurrence network among 79 species.

Table S14. Metabolism data.

Table S15. Differently expressed metabolites in metabolomics data.

Table S16. Table S14 enrichment analysis of metabolomics data.

Table S17. The 35 significantly-different metabolites with more than 4-foldchange.

Table S18. Relationship of remarkable microbial species with significantly-different metabolites.
