# Supplementary figures and images for: Alternation of the gut microbiota in metabolically healthy obesity: An integrated multiomics analysis
Source: Front Cell Infect Microbiol. 2022 Nov 1;12:1012028. doi: 10.3389/fcimb.2022.1012028 (PMC9663839; doi:10.3389/fcimb.2022.1012028)

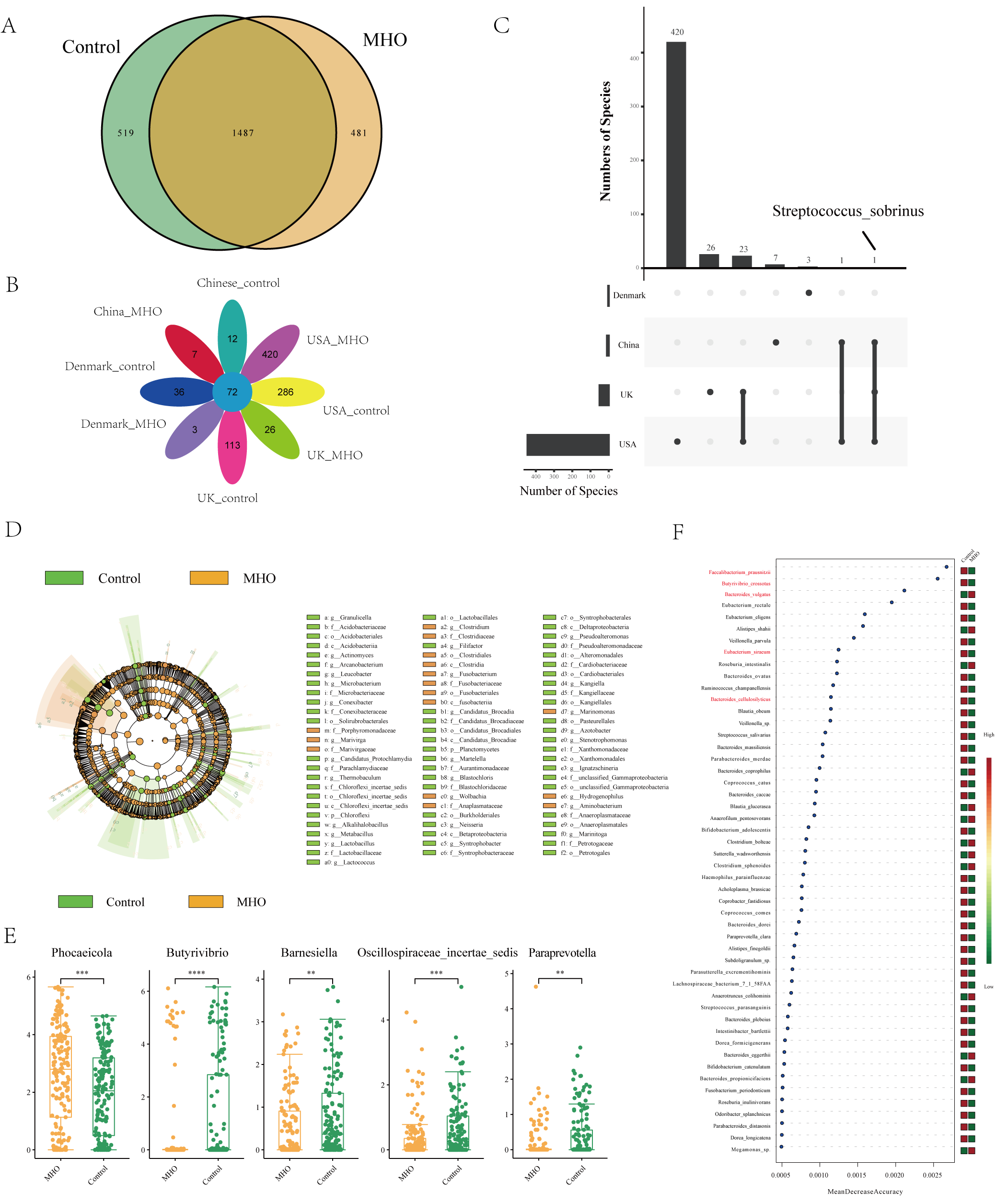

Supplement: Supplementary file 1 [file DataSheet_1.zip › FigureS1.tif]

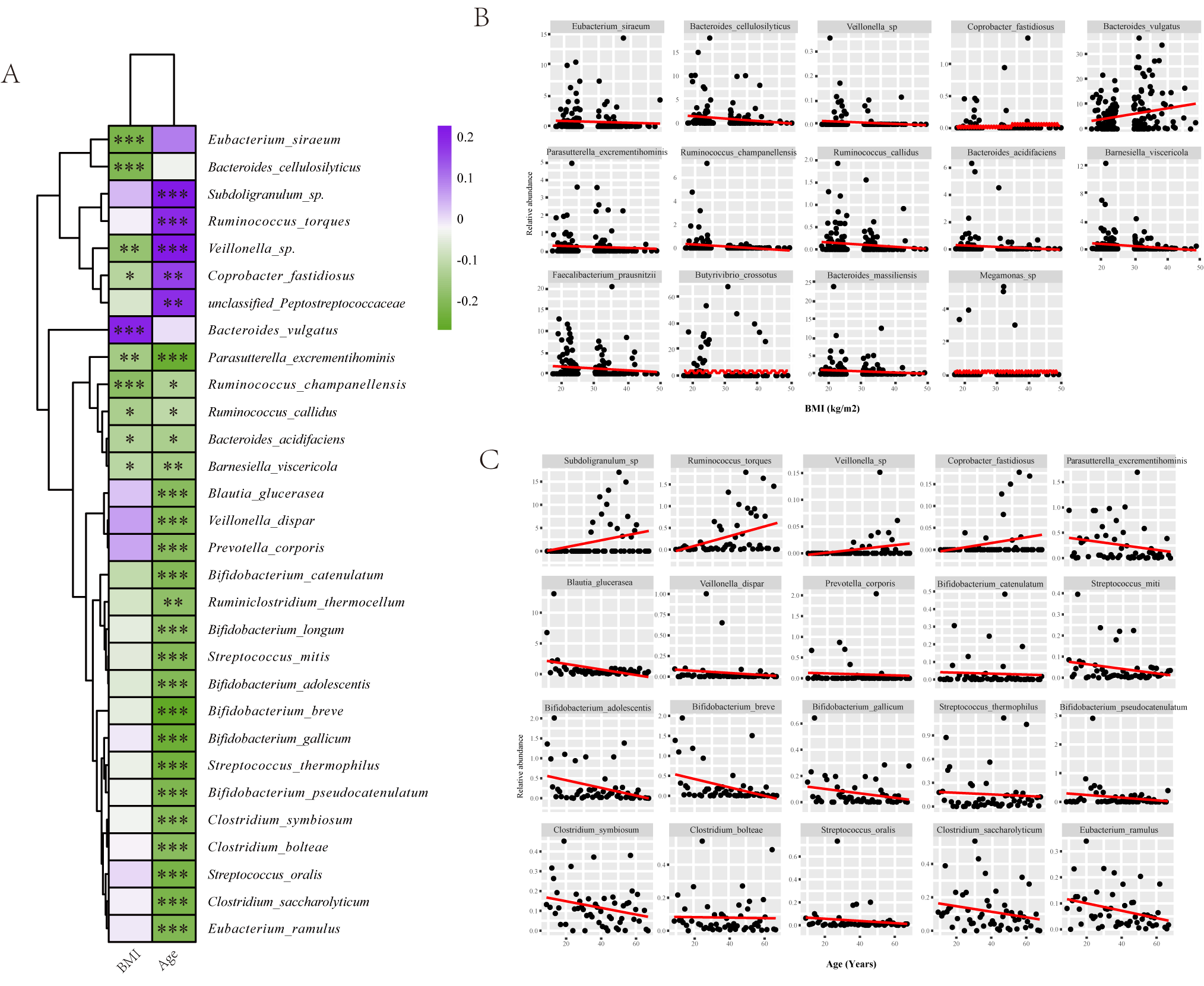

Supplement: Supplementary file 1 [file DataSheet_1.zip › FigureS2_revisedV2.tif]

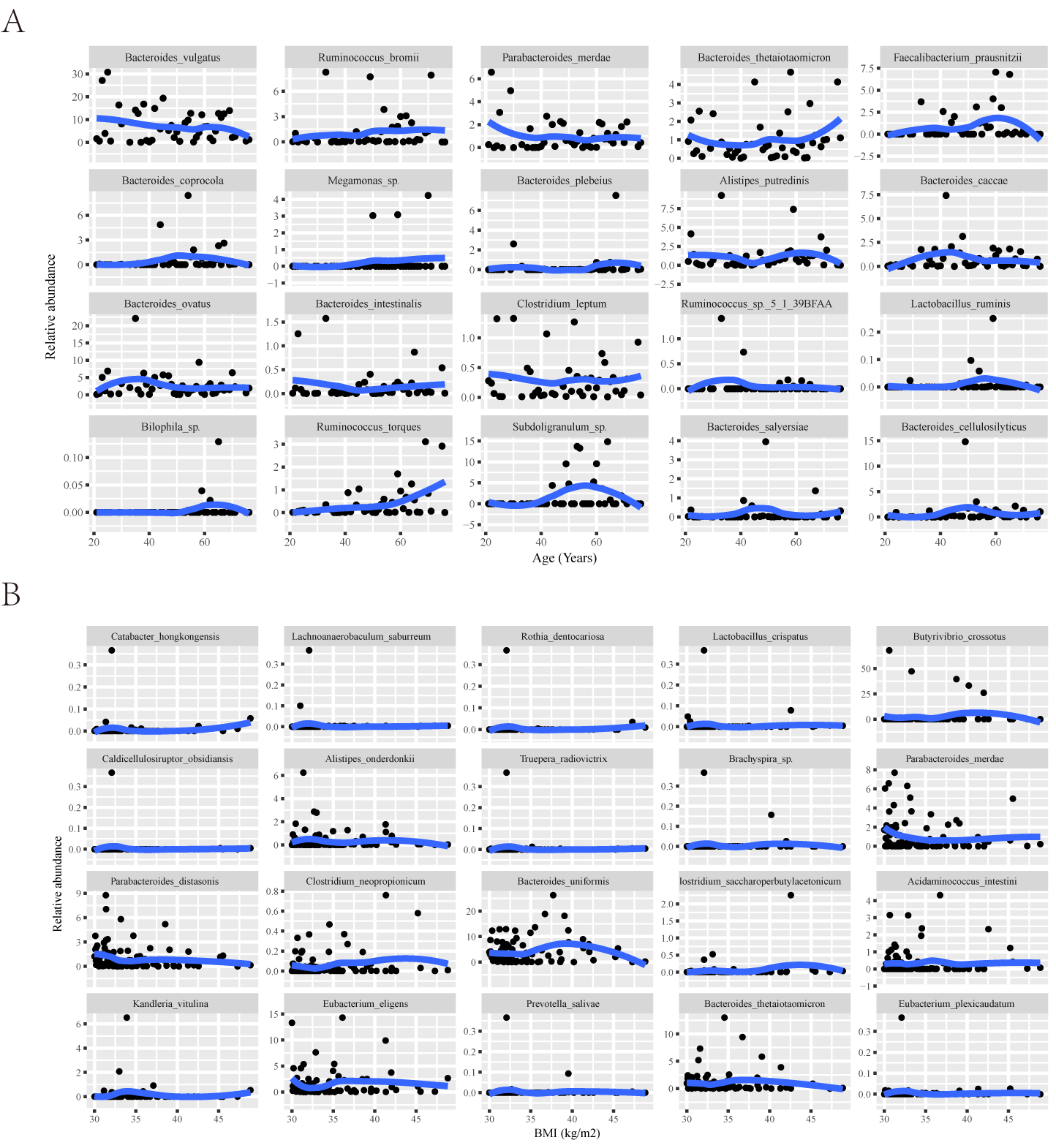

Supplement: Supplementary file 1 [file DataSheet_1.zip › FigureS3_revised.tif]

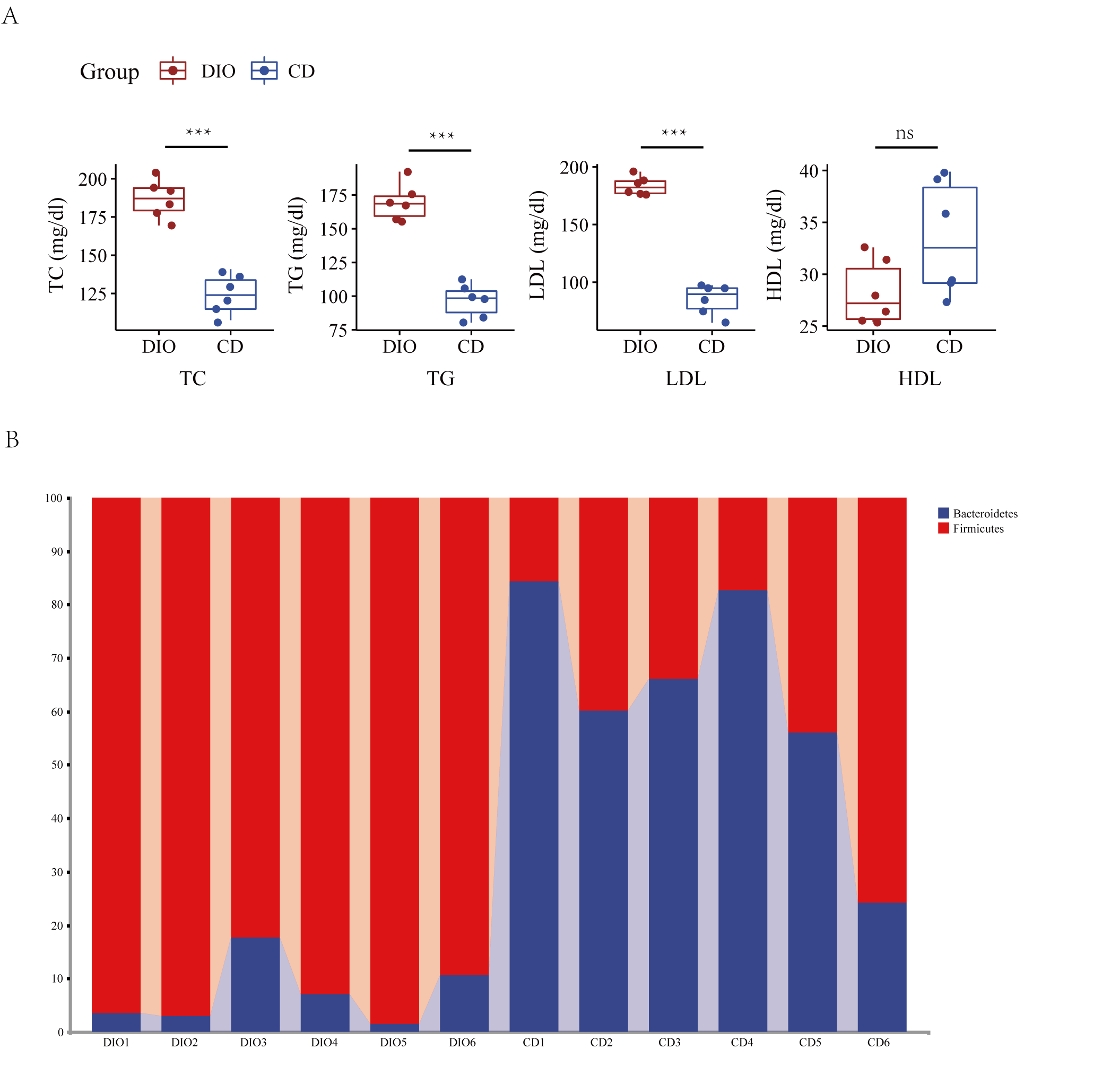

Supplement: Supplementary file 1 [file DataSheet_1.zip › FigureS4_revised.tif]
